# 

**Published:** 1916-01-15

**Authors:** 


### Medical Appointments.

Dr. John MacKeith, M.B. (Glas.), has been appointed medical officer to the tuberculosis department of the Central London Throat, Nose, and Ear Hospital.

At a recent meeting of the Manchester Royal Infirmary Board, Mr. T. Black, M.B., B.Ch., B.A.O., was appointed resident medical officer at the Barnes Convalescent Hospital, Cheadle.

### PASSING EVENTS AND TOPICS.

### Hospital Saturday Fund.

THE total income of this Fund for last year was £36,766, being an increase of £6,636 on the previous year's total.

### Gresham Lectures.

FOUR Gresham Lectures for 1916 on Dietetics will be delivered on January 18, 19, 20, and 21, by Dr. Harry Campbell, M.D., Deputy Gresham Professor of Physic, at Gresham College, Basinghall Street, E.C. The lectures, which are free to the public, will begin each evening at six o'clock.

### Swiss Accommodation for Convalescent Officers.

THE committee of the Public Schools Alpine Sports Club have accepted an offer of the management of the Palace Hotel, Montana, to place that hotel at their disposal, rent free, during the present winter season for the reception of convalescent officers. A charge will be made to each officer of 6s. 6d. per day, to cover cost of food and other expenses, and any balance remaining over will be devoted by the committee to defraying the expenses of necessitous officers.

### Egyptology and Hospital Freemasons.

IT is appropriate that the hieroglyphic of the Egyptian God of Medicine, I-EM HETEP, "the Master of Secrets," should attach itself to the mysteries of the Nosocomia Lodge of Freemasons, founded a few years ago for the convenience of hospital men. His inscription, which means "he who comes in peace," therefore figures on the Past Master's jewel, and was placed on the menu card of the banquet following the installation on January 7 of Mr. Richard Kershaw, the well-known Secretary of the Central London Throat, Nose, and Ear Hospital, as the Master for 1916. The new Master invested the following officers: Messrs. J. Wickliffe Peck, I.P.M.; A. Hayward, S.W.; W. H. Pearce, S.W.; Godfrey H. Hamilton, Acting Treasurer; Stanley Smith, Secretary; J. McKay, J.D.; W. J. Chichele Nourse, D.C.; G. A. Arnaudin, Almoner; A. H. Coughtrey, Assistant Secretary; W. T. Owen and H. W. Burleigh, Stewards. The investment of other officers of the Lodge who are on active service was deferred. An excellent concert under the direction of Mr. F. W. Belchamber, F.R.C.O., followed the banquet.

### Church Collections for the Hospitals.

THE collections in the various places of worship last year for the Metropolitan Hospital Sunday Fund resulted in the receipt of £39,516, being £10,833 more than in 1914. An analysis made by the *National Church* shows that the contributions of the Church of England constitute more than three-fourths of the whole amount collected. The full analysis is as follows: Church of England, £30,296 3s. 7d.; Congregationalists, £1,894 18s. 4d.; Jews, £1,594 15s. 7d.; Presbyterians, £1,248 5s. 9d.; Wesleyans, £1,017 16s. 7d.; Roman Catholics, £781 8s. 8d.; Baptists, £711 15s. 9d.; Church of Scotland, £475 1s. 6d.; Unitarians, £413 2s. 8d.; other denominations and various collections, £1,082 11s. 7d. St. Mark's, North Audley Street, heads the list with a collection of £1,039, while Christ Church, Lancaster Gate, sends £820, and St. Michael's, Chester Square, £691.

### A Surgeon's Precautions Against Infection.

IN a contribution to the *Münchener Medizinische Wochenschrift* Heidenhain states that during thirty-one years in which he has practised surgery he has been injured innumerable times in the course of operations on subjects with sepsis, and has never become infected. His only prophylactic after an injury is to keep the hand and arm in complete rest for twenty-four to forty-eight hours. He relates that once a colleague came to him for a dressing for an autopsy wound, and he ordered immobilisation. Returning from an absence of several days, he found the man dead, the reason, in Heidenhain's view, being that the patient had removed the dressing and very soon experienced a chill. He firmly believes that immobilisation for forty-eight hours after these traumatisms would result in a great reduction of morbidity and mortality among surgeons.

### The Norfolk and Norwich Hospital.

SOME INTERESTING POINTS.

THE quarterly meeting of the governors of the Norfolk and Norwich Hospital took place on January 8, Mr. W. B. Greenfield, J.P., in the chair. The Chairman of the Board of Management (Mr. C. S. Tomes) announced that legacies amounting to £1,300 had been received during the quarter. Further, that a recreation room had been erected in the grounds of the hospital as a voluntary gift from Mr. and Mrs. J. Sancroft Holmes, which would prove itself to be a valuable asset to the institution. The need for such a room for the wounded soldiers has been felt for some time, and will prove doubly welcome during the winter months.

WAR COST OF RESIDENT MEDICAL STAFF.

Mr. Tomes stated that during the past few weeks the whole of the resident medical staff had resigned, having accepted commissions in the R.A.M.C. The hospital, however, had been so fortunate as to secure other medical men who were not eligible for military service. The effects of the war upon the cost of securing a competent resident medical staff have been to increase the present salaries paid by £1,200 more than the sum at which they stood at the outbreak of the war.

MAINTENANCE OF BEDS SCHEME.

The scheme for the maintenance of beds, by which the employees of business firms subscribe £5 per month for each bed, is still a great success, and at the present time about £600 per month is being received from this source. With its aid, and the payments received from the War Office, the hospital has been enabled to face the large financial strain caused by the reception of wounded soldiers.

MR. F. G. HAZELL.

A just and gratifying compliment was paid to the late secretary (Mr. F. G. Hazell) by the Rev. H. W. G. Thursby and Mr. Charles Larking, the only two remaining members of the Board of Management when Mr. Hazell came to the institution in 1900, which was unanimously endorsed by the governors present. The financial statement of the institution to December 31, 1915, the audited accounts relating to which will be presented at the annual general meeting in April next, will be very satisfactory, both as to the amount of work done and the financial aspects of it.

RATES OF SUBSCRIPTION: Twelve months, 6/6; Six months, 4/-; Three months, 2/-, post free to any address in the United Kingdom. Abroad, Twelve months, 8/8; Six months, 5/-; Three months, 2/6, post free.—Address The Subscription Dept., "THE HOSPITAL," The Hospital Building, 28/29 Southampton Street, Strand, London, W.C.

### Manager's Notices.

**All letters on business matters must be addressed to The Manager, "The Hospital," 28 and 29 Southampton Street, London, W.C.**

**Subscriptions**, which may commence at any time, are payable in advance. Cheques and Post-Office Orders should be crossed London County and Westminster Bank, Covent Garden Branch, and made payable to the Manager of THE HOSPITAL.

RATES OF SUBSCRIPTION (payable in advance).

| | | |
|---|---|---|
| UNITED KINGDOM. | | |
| Three Months | (including Postage) | 2s. 0d. |
| Six Months | do. | 4s. 0d. |
| Twelve Months | do. | 6s. 6d. |
| FOREIGN AND COLONIAL. | | |
| Three Months | (including Postage) | 2s. 6d. |
| Six Months | do. | 5s. 0d. |
| Twelve Months | do. | 8s. 8d. |

